# *ANO3* Mutations in Chinese Dystonia: A Genetic Screening Study Using Next-Generation Sequencing

**DOI:** 10.3389/fneur.2019.01351

**Published:** 2020-02-07

**Authors:** Shanglin Li, Lin Wang, Yingmai Yang, Jun Ma, Xinhua Wan

**Affiliations:** ^1^Department of Neurology, Peking Union Medical College Hospital, Peking Union Medical College and Chinese Academy of Medical Sciences, Beijing, China; ^2^Department of Geriatrics, Qilu Hospital, Shandong University, Jinan, China

**Keywords:** ANO3, DYT24, dystonia, Chinese, NGS - next generation sequencing

## Abstract

**Background:** Dystonia-24 (DYT24) is a monogenic autosomal dominant dystonia caused by mutations in the gene ANO3, which has shown phenotypic and genotypic heterogeneity according to previous reports.

**Objective:** To screen and identify ANO3 mutations in a cohort of patients with dystonia in China and to expand the spectrum of DYT24.

**Methods:** This study screened ANO3 mutations in 187 Chinese dystonia patients using next-generation sequencing (NGS). *In silico* investigations were conducted in detected ANO3 variants and co-segregation analysis was carried out if applicable. The effects of identified variants were classified according to the standards and guidelines of the American College of Medical Genetics and Genomics (ACMG).

**Results:** Four different variants were identified in four unrelated dystonia patients, including three missense variants [c.1789G>C (p.V600L), c.182A>C (p.E61A), c.787A>G (p.M263V)] and one splice site change (c.1714-3T>C). The novel missense mutation c.1798G>C (p.V600L), identified in a teenaged girl with generalized dystonia, showed high pathogenicity and was classified as “likely pathogenic” according to ACMG guidelines. Of note, she responded well to deep brain stimulation.

**Conclusion:** Our study helps expand the mutational and clinical spectrum of DYT24 due to ANO3 mutations by further reporting four variants. Rare ANO3 variants appear to represent an uncommon cause of dystonia in China.

## Introduction

Dystonia is a movement disorder characterized by sustained or intermittent muscle contractions causing abnormal often repetitive movements, postures, or both, and is classified based on two distinct axes: clinical features and etiology (1). Etiological categorization based on clinical features is difficult since dystonia has a remarkable degree of phenotypic variability, with frequent overlap with different syndromes. The advent of next-generation sequencing (NGS) technology facilitates the discovery of new pathogenic genes, and different types of dystonia have been identified according to their responsible genes. Dystonia-24 (DYT24) is a monogenic autosomal dominant dystonia due to pathogenic mutations in *ANO3* (NM_031418, encoding for anoctamin 3), which was first identified by Charlesworth et al. (2) with the clinical phenotype of craniocervical dystonia. To date, about 26 different variants (1 from our center in China) have been reported, with a variable clinical spectrum including craniocervical dystonia, myoclonus, limb dystonia, and generalized dystonia, with the age of onset ranging from childhood to the sixth decade of life (3–11). The phenotypic heterogeneity indicates that it is insufficient to just assess *ANO3* mutations in focal dystonia, and emerging sporadic *ANO3* variants raise attention to patients without family history. Thus, screening *ANO3* mutations in dystonia patients without focal dystonia or family histories of dystonia may assist in expanding the diagnostic spectrum of DYT24. With the emerging application of NGS screening technologies in movement disorders, this study aims to screen *ANO3* mutations in inherited or idiopathic dystonia by NGS to identify *ANO3* variants in Chinese patients with dystonia and to expand the phenotypic and genotypic spectrum of DYT24.

## Materials and Methods

### Participants

In this single-center cohort, patients referred to our movement disorders center in the Department of Neurology at Peking Union Medical College Hospital from September 2016 to July 2019 were included according to the following inclusion and exclusion criteria. Inclusion criteria were patients (a) with dystonia as the predominant movement disorder identified by a movement disorder specialist, (b) who were clinically suspected of inherited or idiopathic dystonia with or without family history, and (c) who agreed to genetic testing. Patients meeting the following criteria were excluded: (a) clinical or paraclinical findings suggestive of an acquired cause, or (b) a *TOR1A* or *THAP* mutation identified by Sanger sequencing. Most patients underwent common biochemical testing (e.g., ceruloplasmin) and brain imaging. Demographic clinical data were collected as well as family histories. This study was approved by the ethics committee of Peking Union Medical College Hospital and written informed consent was obtained from all participants or their legal guardians.

### Variant Screening and *in silico* Investigations

Genomic DNA was exacted from blood samples of enrolled patients. Targeted gene capture sequencing designed for movement disorders including 148 genes (53 cases from September 2016 to August 2017) or whole exome sequencing (WES; 134 cases from September 2017 to July 2019) was carried out. Detailed methods have been described in the Supplementary Materials. Only missense, nonsense, splice site, stop-loss, in-frame insertion/deletion, and frameshift variants with a minor allele frequency (MAF) <0.5% (according to reference public databases including 1,000 Genomes Project, Exome Aggregation Consortium, and Genome Aggregation Database) were analyzed. *In silico* analyses were conducted to help evaluate pathogenicity using SIFT (http://sift.jcvi.org), PolyPhen2 (http://genetics.bwh.harvard.edu/pph2), and MutationTaster (http://www.mutationtaster.org). The pathogenicity of identified variants was classified according to the standards and guidelines of the American College of Medical Genetics and Genomics (ACMG) (12).

## Results

### Basic Characteristics

A total of 187 patients were included in the study, with 88 males (47.3%) and 99 females (52.7%). The mean age of onset of the disease was 26.5 years old (range, 1–65). The average age at study inclusion was 31.3 years old (range, 5–67) with a mean disease duration of 4.7 years.

### Identification of *ANO3* Variants

This study identified four variants in *ANO3*, including three missense variants: c.1789G>C (p.V600L), c.787A>G (p.M263V), and c.182A>C (p.E61A) separately in unrelated Patients 1–3, and one splicing variant (c.1714-3T>C) in Patient 4. None of these variants were found in the 1,000 Genomes Project or gnomAD databases. All identified variants were species conservative. Segregation analysis was conducted in two patients, which revealed a *de novo* mutation in Patient 1 and a paternal inheritance in Patient 3. Unfortunately, no family members of the proband were available for further segregation analysis in patients with variants c.787A>G and c.1714-3T>C, either because of social circumstances or because the relatives did not wish to take part in the study. The *in silico* analysis assessments and detailed phenotypic and genotypic results were summarized in Table 1.

**Table 1:** Description of *ANO3* variants identified in 187 dystonia cases, disease characteristics, and predictive pathogenicity.

**Patient no**.	**Age/** **sex**	**Age at Onset** **(years)**	**Movement disorder**	**Dystonia characteristics**					**Family** **history**	**Inheritance**	**Variation**		***In silico*** **analysis**			
				**Classification**	**Face/neck/** **larynx**	**Upper limbs**	**Lower limbs**	**Trunk**			**Gene**	**Protein**	**SIFT**	**Polyphen2**	**Mutationtaster**	**CADD**
1	12/F	11	Isolated dystonia	GENERALIZED	-	Bilateral hands and arms	Bilateral	+	-	De novo	c.1798G>C	p.V600L	Tolerated	Probably damaging	Disease causing	25.7
2	45/F	43	Dystonia, myoclonus	GENERALIZED	Retrocollis with dystonic tremor	Bilateral arms	-	+	-	UK	c.787A>G	p.M263V	Tolerated	Benign	Disease causing	15.13
3	14/F	13	Isolated dystonia	MULTIFOCAL	-	Left hand abnormal posture	Twisting postures in left lower limb, more marked in left foot	_	-	Paternal	c.182A>C	p.E61A	Tolerated	Benign	Polymorphism	14.57
4	19/M	18	Isolated dystonia	FOCAL	-	Writer's cramp in right hand	-	_	-	UK	c.1714-3T>C	-	NA	NA	Disease causing	8.72

*+, positive; –, negative/absent; NA, not applicable; UK, unknown*.

### Case Description

#### Patient 1 c.1789G>C (p.V600L)

A 12-year-old girl presented with abnormal postures in her left lower limb at the age of 11, which were aggravated when walking but almost normal during sleep. She was the only child of her non-consanguineous parents with no family history, or past or perinatal history. The progressing gait problems attracted her parents' attention and they took her to our clinic. Neurological examination showed a normal range of intelligence and generalized dystonia. On enrollment, she demonstrated slight dystonic trunk posturing, abnormal gait, and involuntary movements and abnormal posture in bilateral hands, which were more marked when walking (Supplementary Video 1). Common biochemical testing (e.g., peripheral smear, ceruloplasmin), blood metabolic screening, and brain magnetic resonance imaging (MRI) were unremarkable. WES of the patient and her unaffected biological parents revealed a novel *de novo* heterozygous missense variant in *ANO3*, rated as damaging by PolyPhen2 and MutationTaster. The girl partially responded to benzhexol for 1 month initially. However, over time, the efficacy dwindled, and her gait problems progressed despite multiple titrations in medication. After bilateral globus pallidus interna (Gpi) deep brain stimulation, a substantial improvement of dystonia was obtained, with a self-rated dystonia severity decreasing from 6 (on a scale of 0–10) pre-operation to 2 three months post-operation in a recent follow-up.

#### Patient 2 c.787A>G (p.M263V)

A 45-year-old woman had suffered from abnormal postures and involuntary movements in bilateral upper limbs, neck, and trunk for 2 years. The initial symptoms were myoclonic and dystonic movements in the shoulders, which ceased when lying in bed. Concurrently, her trunk started tilting to the left with progressive retrocollis. She had hyperthyroidism for 20 years with normal thyroid function tests 1 year before disease onset. She denied any significant family history. On examination, she demonstrated generalized dystonia including pronounced retrocollis with dystonic head tremor, and bilateral dystonic arms with occasional myoclonus in shoulders associated with abnormal trunk posturing. Cognitive function, muscle strength, and cerebellar function were normal. Brain imaging was unremarkable. Other tests such as autoimmune tests, metabolic screening, cerebrospinal fluid tests of oligoclonal band or cytology, and paraneoplastic markers were in normal ranges. WES revealed the missense mutation c.970A>G (p.M324V) in *ANO3*, predicted as damaging by PolyPhen2 and MutationTaster separately.

#### Patient 3 c.182A>C (p.E61A)

A 14-year-old girl developed progressive abnormal posturing in the left hand and leg for 1 year. She was diagnosed with scoliosis at 11 years. About 1 year prior to clinic admission, the girl noticed involuntary movement and abnormal posturing of her left hand when playing piano. Two months later, a slight strange posture of her left foot occurred and developed progressively in the following 10 months, resulting in gait problems. Administration of benzhexol showed slight benefits. She was the only child of her non-consanguineous parents with negative family history. A physical exam showed torsion scoliosis and twisting postures of the left foot and hand associated with an abnormal gait. No additional neurological signs such as tremor, spasticity, ataxia, weakness, or Kayser–Fleischer rings were present. Biochemical tests were unremarkable. A brain MRI showed static subtle T2 hyperintensity associated with T1 hypointensity in the right putamen. A novel mutation [c.182A>C (p.E61A)] in *ANO3* was identified by WES and was predicted to be tolerated by SIFT, a polymorphism by MutationTaster, and a CADD (Combined Annotation Dependent Deletion) score of 19.07. Co-segregation analysis revealed that her unaffected father carried the same variant.

#### Patient 4 c.1714-3T>C

A 19-year-old male patient complained of moderate abnormal posture and involuntary movements only in the right hand while writing. Other tasks using the right hand were unaffected. Past history and family history were normal. Upon examination, isolated focal dystonia of writer's cramp in the right hand was present with no additional abnormal neurological signs. Tests including metabolic screening, ceruloplasmin, and brain MRI showed no significant findings. After a trial of levodopa with no benefit, the patient refused to take medicine and began writing using his left hand. WES detected a novel c.1714-3T>C mutation in *ANO3*, predicted as “disease causing” by MutationTaster. As it was a splicing variant, analyses by SIFT and PolyPhen2 were not applicable. Samples of his parents were not available since they lived far away.

## Discussion

DYT24 is an autosomal dominant isolated dystonia with typical clinical features of craniocervical dystonia caused by mutations in *ANO3*, which was first identified by Charlesworth et al. (2). *ANO3* (27 exons) is located in chromosome 11p14.3-11p.14.2, encodes anoctamin 3 (also known as TMEM16C), and is mainly expressed in the striatum, hippocampus, cortex, and dorsal root ganglion (DRG) (13). TMEM16C belongs to the transmembrane protein (TMEM16) family, a group of 10 homologous membrane-spanning proteins, which are related to genetic diseases such as asthma, dystonia, limb-girdle muscular dystrophy, Miyoshi myopathy, Scott syndrome, and cerebellar ataxia (13). Since the identification of the *ANO3* gene in craniocervical dystonia families, subsequent work confirmed the role of this gene in dystonia, which was summarized in Table 2. To date, 26 variants have been reported. In the majority of patients, *ANO3* variants were confirmed in familial torticollis patients. However, sporadic variants with different clinical features of segmental, multifocal, or generalized dystonia have also been reported, with some even accompanied by other neurological signs such as tremor and myoclonus.

**Table 2:** Previously described genetic and clinical characteristics of *ANO3* variants that are likely associated with disease.

**References**	**Variants**		**CADD score**	**Age at onset**	**Family history**	**Dystonia characteristics**		
	**c.DNA**	**Protein**				**Classification**	**Clinical phenotypes**	**Other movement disorders**
Charlesworth et al. (2)	c.2586G>T	K862N	15.89	20–40s	NA	Segmental	CD, OMD	-
	c.161C>T	T54I	21.8	UK	+	NA	ET	-
	c.1480A>T	R494W	29.9	Late 30s	+	Segmental /multifocal	CD, ULD with tremor, 2 LD, 1BSP	-
	c.2053A>G	S685G	16.1	3–25	+	Segmental/focal/ multifocal	CD, LD, tremor in ULD	Myoclonus
	c.1470G>C	W490C	32	Early teens	+	Multifocal	CD, LD, OMD, ULD with tremor	-
	c.-190C>T	-	20.8	Late teens	-	Multifocal	CD, ULD with tremor	-
Zech et al. (3)	c.2497A>G	I833V	15.66	40	-	Focal	CD with tremor	-
	c.2917G>C	G973R	26.3	69	-	Segmental	BSP, OMD	-
Ma et al. (4)	c.2540A>G	Y847C	27.5	41–56	+	Focal/segmental	CD with tremor, BSP, OMD, LD, ULD	-
Blackburn et al. (6)	c.702C>G	C234W	26.1	52	-	Segmental	CD, BSP, OMD	Chorea, vocal tics
Zech et al. (8)	c.674A>G	N225S	16.56	44	-	Segmental	CD, face	N.A.
	c.1528G>A	E510K	34	9	-	Generalized	CD, LD, LLD, ULD, trunk	Myoclonus
	c.1969G>A	A657T	33	12	-	Generalized	LLD, CD, LD, ULD, trunk	Myoclonus
	c.1199G>T	G400V	32	7	-	Segmental	CD, face, LD	-
	c.1387G>A	V463M	24	50	-	Focal	BSP	-
	c.835T>A	Y279N	29.4	65	-	Segmental	CD, face	-
	c.1964_1966du pATA	-	21.2	51	-	Segmental	CD, face	-
Yoo et al. (9, 10)	c.1952G>A	p.S651N	28.4	3	-	Generalized	LLD, ULD, CD, LD, trunk	Myoclonus
	c.860G>A	R287Q	34	54	-	Segmental	CD, UL	-
Olschewski et al. (11)	c.433-2 A>G	I656Nfs*17	24.7	25	-	Focal	Writer cramp	-
	c.982C>T	R328C	25	28/36	UK	Segmental	ULD, dystonic tremor	-
	c.2906G>A	R969Q	32	15/45	-	Focal/segmental	CD, ULD; musician's dystonia	-
	c.1682T>A	V561E	33	3	-	Generalized	CD with tremor, ULD, trunk	-
	c.2894T>G	L965W	27.8	21	-	Segmental	ULD, OMD	-

*CD, cervical dystonia; OMD, oromandibular dystonia; LD, laryngeal dysphonia; LLD, lower limb dystonia; ULD, upper limb dystonia; ET, essential tremor; +, positive; –, negative/absent; UK, unknown; NA, not applicable*.

In our study, *ANO3* variants were found in 2.1% of clinically suspected idiopathic or inherited dystonia patients, which indicated that this type of dystonia might be rare in Chinese patients. However, the exact prevalence of *ANO3* mutations still needs to be evaluated in large sample populations. In addition, we observed heterogeneity in both genotypes and phenotypes in DYT24 caused by *ANO3* mutations in Chinese dystonia patients. In terms of phenotypes, all patients in our study were the only affected ones in their family pedigree. Previous studies have noted two peaks of age of onset in DYT24, one in childhood and one in mid-adulthood (40–49 years). Among the four patients with *ANO3* mutations, three were affected under 18 years of age whereas one developed symptoms in her 40s. Interestingly, only one patient had cervical dystonia, accompanied by dystonia and myoclonus in other parts including the trunk and bilateral upper limbs, which was considered generalized dystonia. One patient presented with generalized dystonia involving the trunk and bilateral lower limbs and hands. Of note, she responded well to deep brain stimulation surgery in line with three previously reported successful operations (8, 9, 11). Similar to a recent study (11), writer's cramp was also observed in one patient with a different missense *ANO3* mutation. Despite a negative past history and the missense mutation in *ANO3* in the patient presenting with dystonia in the left lower limb and hand, we were cautious in diagnosing DYT24 because her brain MRI showed subtle abnormal signals in the contralateral putamen and her unaffected father carried the same variant. However, due to a gradual progression of dystonia despite subtle static abnormal signals in the brain MRI, and a possible reduced penetrance in *ANO3* (5), it is difficult to draw a definite diagnosis of etiology, making further functional studies required.

**Figure 1:**
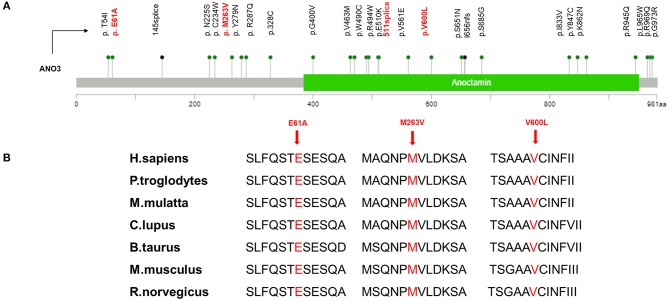
**(A)** Mutation mapper of previously identified *ANO3* mutations related to dystonia were marked in black and variants in our study were marked in red. **(B)** High evolutionary conservation of three missense mutation.

Regarding the genotypes, three missense variants and one splice site change were found in unrelated patients (Figure 1). According to ACMG guidelines, the variant c.1798G>C (p.V600L) was rated “likely pathogenic” because this variant was proven to be *de novo* by co-segregation analysis [strong pathogenic criterion 2 (PS2)], it was absent from population databases [moderate pathogenic criterion 2 (PM2)], and it was predicted to be deleterious by multiple computational methods [supporting pathogenic criterion 3 (PP3)]. The interpretation of the other three variants is difficult and they are rated as “variants of unknown reason” for now, needing additional data such as segregation analysis and functional studies to elucidate their role in DYT24.

However, it remains unclear how *ANO3* mutations cause dystonia at a mechanistic level. Early studies showed that the ANO3 protein (or TMEM16C) functions as a calcium-activated chloride channel, like its family members TMEM16A and TMEM16B (2). However, a recent study by Huang et al. (14) has demonstrated that TMEM16C acts indirectly by regulating sodium-activated potassium channel trafficking in the dorsal root ganglion, which functions in the regulation of pain signaling. Further functional studies are needed to better understand the role of *ANO3* in dystonia.

In summary, our study expands the mutational and clinical spectrum of DYT24 due to *ANO3* mutations in dystonia. Rare *ANO3* variants seem to represent an uncommon cause of dystonia in China. The interpretation of variants in *ANO3* remains difficult, thus demanding further functional studies to better understand the role of *ANO3* in dystonia.

## Data Availability Statement

The raw data supporting the conclusions of this article will be made available by the authors, without undue reservation, to any qualified researcher.

## Ethics Statement

The studies involving human participants were reviewed and approved by Institutional Review Board of Peking Union Medical College Hospital. Written informed consent to participate in this study was provided by the participants' legal guardian/next of kin. Written informed consent was obtained from the individual(s), and minor(s)' legal guardian/next of kin, for the publication of any potentially identifiable images or data included in this article.

## Author Contributions

SL: conception of the work, data acquisition, statistical analysis, and writing of the first draft. XW: design and organization of the work, manuscript review, and critique. LW, YY, and JM: data acquisition, manuscript review, and critique.

### Conflict of Interest

The authors declare that the research was conducted in the absence of any commercial or financial relationships that could be construed as a potential conflict of interest.
