# Supplementary material for: ANO3 Mutations in Chinese Dystonia: A Genetic Screening Study Using Next-Generation Sequencing
Source: Front Neurol. 2020 Feb 7;10:1351. doi: 10.3389/fneur.2019.01351 (PMC7020335; doi:10.3389/fneur.2019.01351)
Supplement: Supplementary file 1 [file Data_Sheet_1.DOCX]

Video 1. Patient 1 with a de novo *ANO3* missense variant c.1789C (p.V600L)

Dystonia in bilateral lower limbs and hands were observed.

**Supplemented Methods**

Genetic testing: Collect 4ml venous blood from the individual to Ethylenediaminetetraacetic acid (EDTA) – anticoagulated tube. Extract genomic DNA using FlexiGene DNA kits (Qiagen, product number 51206). The content of DNA was assayed using an ultramicro spectrophotometer Nanodrop 2000 (Thermo Fisher Scientific) using Agilent SureSelect XT Human All Exon V6 (Agilent). DNA was fragmented into 150-300 bp length fragments using an ultrasonoscope (Covaris S2, USA). The DNA fragments were then processed by end-repairing, A-tailing and adaptor ligation, PCR amplification, and enriched by the whole-exome panel. Paired-end sequencing was performed on llumina HiSeq X-ten platform to provide a mean sequence coverage of more than 100X and a coverage of at least 20X in more than 95% target areas. The generated data was then processed using bioinformatic tools.

For the quality control, a few unqualified sequences from the primary data included 3′-/5′- adaptor sequences and low-quality reads were removed by the Cutadapt and FastQC, respectively. The remaining sequences were termed as clean reads for further analysis. The clean reads were then mapped to the reference human genome (UCSC hg19) using the BWA (Burrows Wheeler Aligner). Duplicated reads were marked and removed using Picard Tools by default parameters. Short read alignment and variants visualization were performed by the IGV (Integrative Genomics Viewer). SNVs and indels were identified using the GATK (The Genome Analysis Toolkit). Subsequent annotation was performed using ANNOVAR with various descriptive information, including function implication (gene region, functional effect, mRNA GenBank accession number, amino acid change, etc.) and allele frequency in population databases (1000 Genomes, ExAc and gnomAD). Only missense, nonsense, splice-site, in-frame insertion/deletion, and frameshift variants with population minor allele frequency (MAF) < 1% were selected for further assessment. Previously identified SNPs were determined using the NCBI dbSNP. Known disease-causing mutations were identified from the professional version of Human Gene Mutation Database (HGMD, http://www.hgmd.org) or from mutations previously reported in the literature. Unreported missense mutations were predicted by SIFT (http://sift.jcvi.org) , PolyPhen-2 (http://genetics.bwh.harvard.edu/pph2), MutationTaster (http://www.mutationtaster.org) and CADD (http://cadd.gs.washington.edu). The phenotypes of the screened genes and the inherited modes were also taken into consideration in order to further exclude irrelevant genes. Then the variants left were verified by Sanger sequencing and tested in other family members and 100 Chinese unrelated healthy individuals. Interpretations of the variants were according to the American College of Medical Genetics and Genomics (ACMG) Standards and Guidelines. All the variants were categorized into pathogenic, likely pathogenic, uncertain significance, likely benign and benign.
